# NanR, a Transcriptional Regulator That Binds to the Promoters of Genes Involved in Sialic Acid Metabolism in the Anaerobic Pathogen *Clostridium perfringens*

**DOI:** 10.1371/journal.pone.0133217

**Published:** 2015-07-21

**Authors:** Blair Therit, Jackie K. Cheung, Julian I. Rood, Stephen B. Melville

**Affiliations:** 1 Department of Biological Sciences, Virginia Tech, Blacksburg, Virginia, United States of America; 2 Department of Microbiology, Monash University, Clayton, VIC, Australia; Beijing Institute of Microbiology and Epidemiology, CHINA

## Abstract

Among many other virulence factors, *Clostridium perfringens* produces three sialidases NanH, NanI and NanJ. NanH lacks a secretion signal peptide and is predicted to be an intracellular enzyme, while NanI and NanJ are secreted. Previously, we had identified part of an operon encoding NanE (epimerase) and NanA (sialic acid lyase) enzymes. Further analysis of the entire operon suggests that it encodes a complete pathway for the transport and metabolism of sialic acid along with a putative transcriptional regulator, NanR. The addition of 30 mM N-acetyl neuraminic acid (Neu5Ac) to a semi-defined medium significantly enhanced the growth yield of strain 13, suggesting that Neu5Ac can be used as a nutrient. *C*. *perfringens* strain 13 lacks a *nanH* gene, but has NanI- and NanJ-encoding genes. Analysis of *nanI*, *nanJ*, and *nanInanJ* mutants constructed by homologous recombination revealed that the expression of the major sialidase, NanI, was induced by the addition of Neu5Ac to the medium, and that in separate experiments, the same was true of a *nanI-gusA* transcriptional fusion. For the *nanI* and *nanJ* genes, primer extension identified three and two putative transcription start sites, respectively. Gel mobility shift assays using purified NanR and DNA from the promoter regions of the *nanI* and *nanE* genes showed high affinity, specific binding by NanR. We propose that NanR is a global regulator of sialic acid-associated genes and that it responds, in a positive feedback loop, to the concentration of sialic acid in the cell.

## Introduction

*Clostridium perfringens* is an anaerobic, Gram-positive pathogen of humans and animals and produces at least 19 different toxins and extracellular hydrolytic enzymes, including α-toxin (PLC or CPA) and perfringolysin O (PFO), which are the major virulence factors of *C*. *perfringens* gas gangrene infections [1–3]. The many extracellular enzymes produced by *C*. *perfringens* include sialidases, or neuraminidases, which are glycohydrolases that cleave the terminal sialic acid from sialoglycoconjugates [4]. *C*. *perfringens* strains produce up to three sialidases: two large secreted sialidases, NanI and NanJ, and a small (43 kDa) intracellular sialidase, NanH [5–8]. Genome sequencing showed that the majority, but not all, strains carry all three sialidase-encoding genes [9]. However, the gas gangrene-causing isolate, strain 13, has the *nanI* and *nanJ* genes, but lacks the *nanH* gene [8], while strain, SM101, an electroporation-competent derivative of the food poisoning strain NCTC 8798 [10], has only the *nanH* gene [11]. Previous studies showed that NanI was responsible for most of the sialidase activity produced by strain 13 derivatives and that neither enzyme was essential for disease in the mouse myonecrosis model [12].

Sialidases can be metabolically useful by supplying substrates that can act as a source of both carbon and nitrogen [4, 13]. Previous reports [14, 15] have described the cloning and sequencing of a *C*. *perfringens* locus that encodes the genes for a putative N-acetylmannosamine-6-P epimerase (*nanE*) and a sialic acid lyase (*nanA*), both of which are involved in the breakdown and utilization of sialic acid [14]. We identified the promoter for the *nanE*/*nanA* operon and demonstrated, *via* primer extension and Northern blot experiments, that transcription of the operon was induced by adding sialic acid to the medium [14].

Studies on the regulation of sialidase production in *C*. *perfringens* showed that extracellular sialidase enzyme activity was induced by the addition of free sialic acid to the medium [16], which makes it probable that a specific regulatory system responds to sialic acid to regulate transcription of the sialidase-encoding genes. Multiple proteins are involved in regulating sialic acid-associated gene expression. The VirSR two-component signal transduction system positively regulates the transcription of the *nanI*, *nanJ* and *nanEA* genes [17], although the signal for regulating the VirSR system is believed to be related under certain conditions to quorum sensing [18] and is not specific for the presence of sialic acid in the medium. In addition, the orphan response regulator RevR directly or indirectly regulates *nanI* and *nanJ* transcription in a negative and positive manner, respectively, although this process also does not seem to be specific to sialidase-encoding genes, since more than 100 genes were differentially expressed in a *revR* mutant [19]. Abe *et al*. [20] found that an RNA-binding protein called Tex (CPE2168) also affected mRNA levels of *nanJ* in a non-sialic acid dependent manner and in another study *nanI* expression was shown to be negatively regulated by a heterocomplex protein pair, CPE1446 and CPE1447, also in a non-sialic acid dependent manner [21]. Finally, a mutation introduced into the *reeS* gene, which encodes an orphan sensor histidine kinase, led to down regulation of *nanI* and *nanJ* transcription and enzyme synthesis in strain 13 [22], but again this regulation occurs in the absence of sialic acid in the medium. Therefore, a sialic acid-specific transcriptional regulator remains to be discovered in *C*. *perfringens*.

Transcriptional regulators that do respond to sialic acid have been characterized in both Gram-negative and Gram-positive bacteria. These regulators tend to fall into two groups of proteins: one group, which contains members of the GntR family of transcription regulators, has been found mostly in enteric bacteria, and the other group belongs to the RpiR family of transcriptional regulators, some of which have been shown to bind phospho-sugars, which are intermediates in sialic acid metabolism [23–25].

In this report, we identified an operon encoding all of the enzymes required for sialic acid metabolism and generated *nanI-* and *nanJ*- mutants in strain 13 to determine which sialidases are regulated by sialic acid. We also investigated the regulation of *nanI* and *nanJ* in response to exogenous sialic acid, and identified an RpiR-family transcriptional regulator, NanR, that was encoded by the *nanE*/A operon and which bound with high affinity and specificity to the *nanI* and *nanE* promoters.

## Materials and Methods

### Bacterial strains and growth conditions

Bacterial strains and plasmids used in this report are listed in Table 1. Luria Bertani broth (LB) (10 g tryptone, 5 g NaCl, 5 g yeast extract) was used to grow *E*. *coli*. To grow *C*. *perfringens*, anaerobic PGY medium (30 g proteose peptone, 20 g glucose, 10 g yeast extract, and 1 g sodium thioglycolate per liter) or PY medium (PGY lacking glucose) was prepared and stored in a Coy anaerobic chamber (Coy Laboratory Products, Inc.), as previously described [10], with 1% agar added for plates as needed. To determine if *C*. *perfringens* strains could grow with N-acetyl neuraminic acid (Neu5Ac) as the major carbon source, the cells were inoculated into a semi-defined media (0.1% yeast extract, 50 mM NaPO_4_ (pH 7.0), 19.79 mg/L MnCl_2_·4H_2_O, 122 mg/L MgCl_2_·6H_2_O, 29.4 mg/L CaCl_2_·2H_2_O, 11.5 mg/L ZnSO_4_·7H_2_O, 0.025 mg/L CuSO_4_·5H_2_O). The yeast extract was added because *C*. *perfringens* strains are multiple auxotrophs, requiring up to 20 different amino acids and vitamins [26, 27]. Neu5Ac (30 mM) (Pfaltz and Bauer Chemical Co.) was added as indicated. For growth curves, strain 13 was grown overnight at 37°C in an anaerobic chamber in the semi-defined medium with 10 mM glucose (OD_600_ of the overnight cultures were ~0.43) and inoculated at a 1:50 dilution into screw-capped glass tubes containing semi-defined medium. After inoculation, the tubes were left in the anaerobic chamber for 1–2 hours. Teflon tape was wrapped around the top of the tube, the screw cap was tightened, and the tubes removed from the anaerobic chamber and incubated at 37°C. At the indicated times, the OD_600_ was measured using a Bausch and Lomb Spectronic 20 spectrophotometer.

**Table 1:** Strains, plasmids and primers.

Strains/Plasmid	Relevant characteristics	Source or reference
***E*. *coli***		
DH10B	F- *mcrA* Δ(*mrr*-*hsdRMS*-*mcrBC*) ΦΔ*lacZ*ΔM15 Δ*lacX74 deoR recA1 araΔ139* Δ(*ara*, *leu*)7697 *galU galK* λ- *rpsL endA1 nupG*	[28]
JM107	*endA*’ *gyrA96 thi hsdR17 supE44 relA1* λ-Δ(*lac* ^-^ *proAB*) F’ *traD36 proAB lac19 lacZ* ΔM13	[29]
BL21(DE3) pRIL	*F* ^*–*^ * ompT gal dcm lon hsdS*_*B*_ *(r*_*B*_^*-*^ * m*_*B*_^*-*^ *) λ(DE3 [lacI lacUV5-T7* gene *1 ind1 sam7 nin5])* pRIL(cm^R^) carries rare low G-C codon tRNAs: *argU* (AGA, AGG), *ileY* (AUA), *leuW* (CUA)	Stratagene
***C*. *perfringens***		
13	Gas gangrene strain	[30]
AH1	13*bglRermB*	[31]
SM101	High frequency transformable derivative of NCTC 8798	[10]
BT1	13*nanIermB*	This study
BT2	13*nanJcatP*	This study
BT3	BT1*nanJcatP*	This study
**Plasmids**		
pSK-	*E*. *coli* cloning vector	Stratagene
pGEM-T Easy	*E*. *coli* cloning vector	Promega
pET24a(+)	*E*. *coli* expression vector	Novagen
pSM218	Transcription vector, *catP* ^+^	[10]
pSM300	*C*. *perfringens* suicide vector, *ermB* ^*+*^	[3]
pJIR750	*E*. *coli*-*C*. *perfringens* shuttle vector, *catP* ^+^	[32]
pJV50	*C*. *perfringens* suicide vector, *catP* ^*+*^	This study
pSM230	pSM218Ω*nanI* promoter region from strain 13	This study
pSM240	pSM218 with polylinker of pBluescript SK+ (Stratagene corp.)	[31]
pDOB18	pSM300Ω, internal fragment of *nanI* from strain 13	This study
pBT5	pJV50Ω, and internal fragment of *nanJ* from strain 13	This study
pSM323	pGEMT-Easy with wild-type *nanR* from strain 13	This study
pSM324	pET24a(+) with wild-type *nanR* from strain 13	This study
**Primers**	**Sequence (5’ to 3’)**	
OSM90	TTTAAAGCATGCGTACACTTAGAGTTTTTAAAATGAG	
OSM91	AAAATTAATCTAGACCTTTGTAATTCATTTTAC	
OSM249	CAGTAAGAGGTACCATATGGGAATATTAG	
OSM250	GATACAAGAATTCTCATTAAGAATTGCACTAG	
OBT34	CAAGTGTAGCTATAATTTTTTTACTTTTCACTAGCTC	
OJV38	AGTGTCTCTAGAGCCTACGGGG	
OJV45	GGGTAACAAAAAACACCGTATTTCTACG	
ODOB11	CCTTAATGGTACAATCATAAAAGAAGTTAAAGATA	
ODOB12	ATAAGGTTCTAGAATTTATTATTTTTCCATTTTC	
OBT21	GGGGAAACTAAGGCGCCTGCAGAGG	
OBT22	GCCTGCCCAAGCTTCCTCG	
JRP5970	CTTTCAATAATTACCCCTCC	
JRP5971	GACCTTATATTATGTATACG	
JRP5972	AGTTAAAAGCAATTAAAAAAAC	
JRP5973	ATGAATATACTTCTTGAAATGC	
JRP5974	ATATTTTGATACATGCTC	
JRP5975	CATTTTATCACCTTTTTTC	
JRP5976	AGATAACTATGAAAATGG	
JRP5977	TATTATTGTCCTTTATTTCAAG	

### Plasmid constructs

To determine if the region upstream of *nanI* can function as a promoter, a PCR product, amplified using primers OSM90 and OSM91 and containing 664 bp of the intergenic region between *nanI* and the upstream gene [5], was placed upstream of the *gusA* gene in pSM218 [14] to create pSM230 (Table 1). pSM218 is an *E*. *coli*-*C*. *perfringens* shuttle vector containing a promoterless *cpe*-*gusA* gene fusion, which retains the ribosomal binding site and first 13 amino acids of the *cpe* gene, preceded by a polylinker region and four tandem terminators [14]. The *nanI*-*gusA* fusion contained the entire promoter region and the first three codons of the *nanI* structural gene. The resulting plasmid, pSM230, was introduced into *C*. *perfringens* strain AH1 [31] by electroporation, as previously described [33].

### Primer extension experiments

To determine the transcription start sites for *nanI* and *nanJ*, strain 13 was grown to mid-log phase in either PY or PY supplemented with 1 mg/ml sialic acid and total RNA was extracted from the cells using the TRIZOL reagent (Invitrogen), as previously described [33]. Two different methods were used to map the transcription start sites. For *nanI*, twenty **μ**g of RNA was used as a template for primer extension reactions, using the Promega Primer Extension System kit in accordance with the manufacturer’s instructions. The primer used for *nanI*, OSM91 (Table 1) was designed to anneal to the first 10 codons and 6 bp upstream of the *nanI* structural gene. The DNA sequencing ladder was made using ^35^S-labeled dATP and OSM91, according to the United States Biochemical Co. Sequenase version 2.0 sequencing protocol. For *nanJ*, primer extension was done using 1 **μ**g of RNA with the primer OBT34 (Table 1), which includes the first 10 codons of *nanJ*, tagged with 6-carboxyfluorescein (6-FAM) on the 5’-end. Fluorescently labeled oligonucleotide extension (FLOE) was performed as previously described [34].

### Construction of nanI and nanJ mutants

To create the *nanI* mutant, BT1, a *C*. *perfringens* suicide vector containing an internal fragment of the *nanI* structural gene was constructed. A 1,091 bp internal fragment of the *nanI* gene was amplified with oligonucleotides ODOB11 and ODOB12 and ligated to pSM300, a *C*. *perfringens* replication deficient plasmid [35], to form pDOB18. The integration vector pSM300 encodes the *ermB* gene, thereby conferring erythromycin resistance.

A *C*. *perfringens* suicide vector containing an internal region of the *nanJ* structural gene was constructed and used to make the *nanJ* mutant, BT2 (Table 1). A 1,416 bp internal fragment of the *nanJ* gene was amplified by PCR with oligonucleotides OBT21 and OBT22, which are located 1,090 bases downstream of the *nanJ* start codon, and 1,016 bp upstream of the *nanJ* stop codon, respectively. The PCR product was ligated to the pGEM-T Easy vector (Promega Inc.) as per the manufacturer’s instructions. The insert was then digested with *Hin*dIII and *Pst*I, giving a 1.4 kb fragment, which was ligated into pJV50, to form pBT5. pJV50 is a suicide vector that confers chloramphenicol resistance. It was constructed by blunt end ligation of the *catP* gene from pJIR750 [32], which was amplified by PCR using primers OJV38 and OJV45, into *Dra*I/*Ssp*I digested pSK-.

pDOB18 and pBT5 were used to transform *E*. *coli* strain JM107 and plasmid DNA was purified by cesium chloride gradient ultracentrifugation [36]. The resulting plasmids were used to transform overnight cultures of *C*. *perfringens* strain 13 by electroporation with 4 mm-gap cuvettes and 60 **μ**g of plasmid DNA. Potential mutants were selected on Brain Heart Infusion (BHI) agar plates supplemented with either 30 **μ**g/ml erythromycin (*nanI*) or 20 **μ**g/ml chloramphenicol (*nanJ*) and designated as BT1 and BT2, respectively. To construct the *nanInanJ* double mutant BT3, strain BT1 was transformed with pBT5, selecting for chromosomal insertions on BHI agar plates with 30 **μ**g/ml of erythromycin and 20 **μ**g/ml of chloramphenicol. All mutations were confirmed by Southern blot analysis (S1 Fig).

### Purification of NanR

The *nanR* gene (*CPE0189*) was amplified by PCR using primers OSM249 and OSM250 and the product was cloned into the PCR cloning vector, pGEM-T Easy to form pSM323. pSM323 was digested with *Nde*I and *Eco*RI (these sites were part of the OSM249 and OSM250 primer sequences) and ligated into *Nde*I/*Eco*RI digested pET24a(+), to create pSM324. This process placed a His(6) tag at the C-terminus of the encoded protein. To overexpress the NanR protein, *E*. *coli* BL21(DE3) C43 pRIL cells containing pSM324 were cultured at 37°C, with shaking, in LB broth supplemented with kanamycin (100 **μ**g/ml) and chloramphenicol (30 **μ**g/ml). Protein expression was induced with IPTG at a final concentration of 0.5 mM, when the OD_600_ of the culture reached 0.4. Induction was carried out overnight at 19°C with shaking. Harvested cells were resuspended in NanR buffer (100 mM sodium phosphate, 150 mM NaCl, pH 7.5), lysed by sonication, and the resulting lysate cleared by centrifugation at 18,000 x *g* for 20 min at 4°C. His-tagged NanR was purified by nickel affinity chromatography, where the column was washed with IMAC-25 buffer (100 mM sodium phosphate, 150 mM NaCl, 25 mM imidazole, pH 7.5), followed by IMAC-100 buffer (100 mM sodium phosphate, 150 mM NaCl, 100 mM imidazole, pH 7.5). NanR was eluted using a gradient from 100 mM imidazole to 500 mM imidazole, and subsequently loaded onto a Hiload 16/60 Superdex 200 prep grade column (GE Biosciences) in NanR buffer. Eluted samples were analyzed by SDS-PAGE, and fractions containing purified NanR were pooled. An image showing the purity of NanR protein during the Superdex column chromatography is included in the Supplemental Information, S2 Fig. Protein concentration was determined using the BioRad protein assay as per the manufacturer’s instructions.

### Gel mobility shift experiments

The 401 bp *nanI* and 235 bp *nanE* probes were PCR amplified with JRP5972/JRP5973 and JRP5974/JRP5975 primer pairs (Table 1), respectively. The probes for *nanJ* (191 bp) and *nanR* (332 bp) were PCR amplified using JRP5970/JRP5971 and JRP5976/JRP5977, respectively. PCR products were purified and end-labeled with digoxigenin-11-ddUTP using the DIG gel shift kit 2^nd^ generation (Roche) in accordance with the manufacturer’s instructions. Gel mobility shift binding reactions consisted of 7.5 fmol of DIG-labeled probe, varying concentrations of NanR (12.5 nM, 25 nM, 50 nM, 100 nM, or 200 nM), binding buffer (20 mM HEPES, pH 7.6, 1 mM EDTA, 10 mM (NH_4_)_2_SO_4_, 1 mM DTT, 0.2% (w/v) Tween^20^, 30 mM KCl), 1 μg of poly [d(I-C)] and 0.1 μg of poly-lysine. Competition binding reactions contained 100 nM of NanR, binding buffer, poly [d(I-C)], poly-lysine as above, as well as 10X, 50X or 100X excess unlabeled *nanI* or *nanE* probes, which equated to 75 fmol, 375 fmol or 750 fmol of unlabeled DNA. These competition binding reactions were pre-incubated at room temperature for 10 min before the addition of 7.5 fmol of DIG-labeled probe. All binding reactions were incubated at room temperature for 20 min prior to separation by electrophoresis at 100 V in a 5% native 0.5X TBE polyacrylamide gel at 4°C. Target DNA was transferred onto positively charged nylon membranes (Roche) using the BioRad mini trans-blot transfer cell at 80 V for 1 h at 4°C in 0.5X TBE buffer. DIG-labeled DNA probes were detected with CSPD (Roche) as outlined by the manufacturer.

### Sialidase and β-glucuronidase enzyme assays

For sialidase assays, *C*. *perfringens* cultures were grown overnight in an anaerobic chamber in 5 ml of PY media, in either the presence or absence of 1 mg/ml of sialic acid (N-acetylneuraminic acid) or the equivalent volume of sterile water as a control. The overnight cultures were pelleted by centrifugation in a IEC table top clinical centrifuge (4,100 x g) at room temperature and the supernatants were removed. The measurement of NanI sialidase activity in the supernatants was performed as previously described [6] with the following modification: the fluorogenic substrate 4-methylumbelliferyl-α-D-N-acetylneuraminic acid (MU-Neu5Ac; Sigma) was diluted to a final concentration of 0.1 mM. To determine the amount of released substrate, the fluorescence generated was compared to a standard curve created using pure methylumbelliferone (MU) (Sigma-Aldrich). The released MU was quantified using a Tecan SPECTRAfluor Plus plate reader. β-glucuronidase assays in *C*. *perfringens* were done as previously described [33].

### Statistics

Statistical analyses (student's two-tailed t-tests) were done using InStat 3 software (Graphpad Inc.). For all statistical analyses, *P* values of <0.05 were considered significant.

## Results

### Assignment of gene product functions in the major nan locus

**Fig 1:**
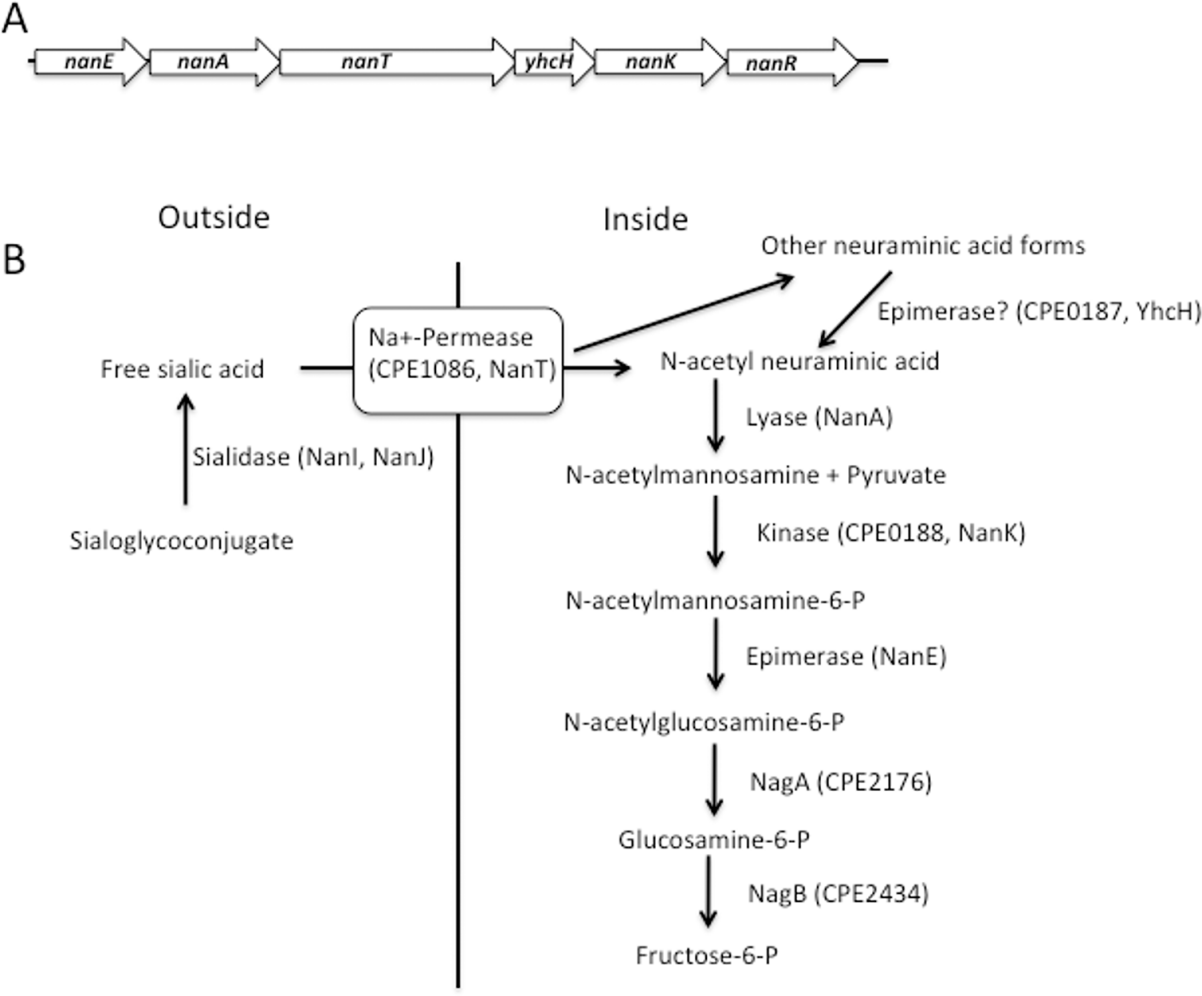
Genes encoding enzymes involved in sialic acid metabolism in *C*. *perfringens*. **(A)**. Structure of the putative *nanEAT*-*yhcH*-*nanKR* operon in strain 13. **(B)**. Proposed pathway of sialic acid metabolism in *C*. *perfringens* strain 13 in relation to the gene content in panel A.

In a previous report, we identified part of an operon from strain NCTC 8798 that contained the *nanE* and *nanA* genes, and part of the gene encoding a putative Na^+^-dependent permease [14]. At approximately the same time, other workers cloned and characterized a similar region from *C*. *perfringens* strain A99 [15]. The lack of a published genome sequence at that time delayed further analysis of the downstream genes, but this is now possible with the release of >12 *C*. *perfringens* genome sequences [9]. In Fig 1A, the gene order for the operon that begins with *nanE* is shown, while the corresponding function of the encoded gene products in the sialic acid metabolism pathway is shown in Fig 1B. Strain 13 has genes encoding the NanI and NanJ extracellular sialidases shown in Fig 1B (but lacks a *nanH* gene [8]); however, these genes are located at sites separate from the putative *nanEAT*-*yhcH*-*nanKR* operon shown in Fig 1A. The *nan* operon synteny is conserved throughout all the strains of *C*. *perfringens* that have been sequenced thus far [9].

The gene encoding a proposed sialic acid permease, *nanT*, is next in the sequence after the *nanA* gene. BLAST P [37] analysis of the NanT protein predicts it is most likely a sodium:solute symporter with multiple homologs in Gram-positive species. The *C*. *perfringens* NanT protein has 32% amino acid sequence identity to over 84% of NanT from *Staphylococcus aureus*, which has been shown to be required for growth on Neu5Ac [25], indicating that it may function as a sodium:neuraminic acid symporter in *C*. *perfringens*. Based on BLAST P searches, the next gene has been tentatively assigned to encode a homolog of the YhcH protein from *E*. *coli*. It does not have a known function, but has been proposed, based on structural and genetic studies [38], to be an epimerase that converts other forms of sialic acid to the preferred substrate of NanA, Neu5Ac (Fig 1B). The *nanK* gene product is predicted to be a sugar kinase using BLAST P searches and shows 31% amino acid sequence identity to over 95% of the NanK protein from *S*. *aureus*. The last gene in the operon encodes an ortholog of the RpiR-family of transcriptional regulators, which bind phosphosugars as part of their regulation of metabolic pathways [23–25]. We have named this protein NanR. Other enzymes putatively involved in the conversion of N-acetylglucosamine-6-P to fructose-6-P, NagA and NagB, are not specific for Neu5Ac metabolism and are encoded by genes in different loci on the *C*. *perfringens* chromosome (Fig 1B).

### Sialic acid increases the growth rate and yield of *C*. *perfringens* in a medium lacking added carbohydrates

**Fig 2:**
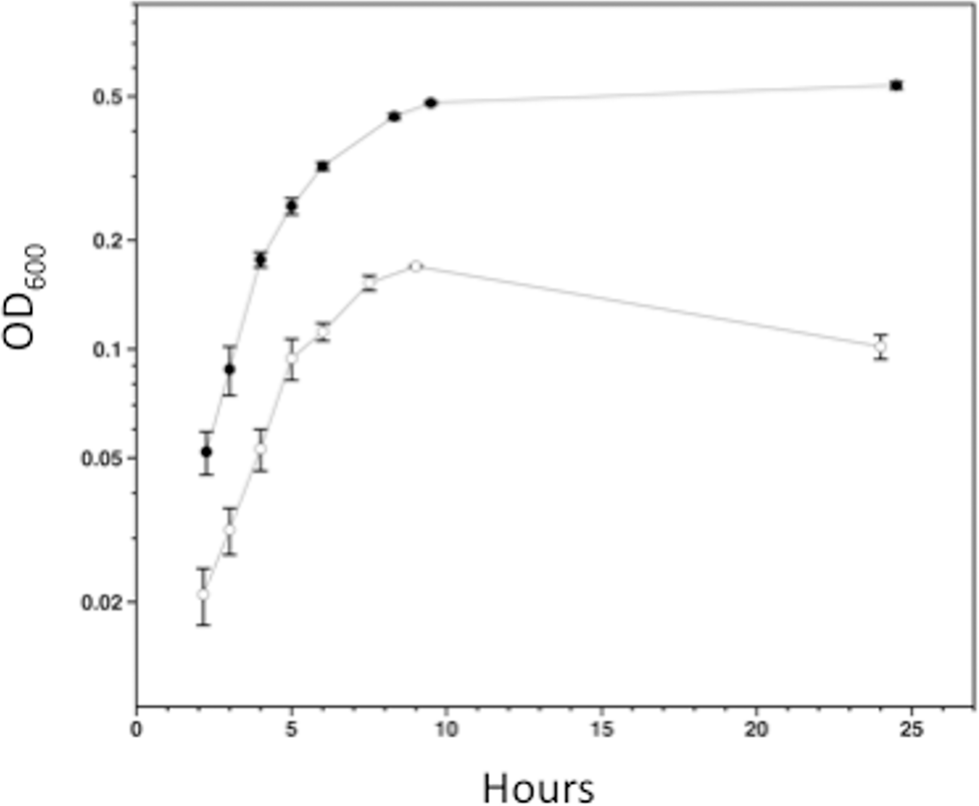
Growth of *C*. *perfringens* strain 13 in semi-defined medium in the presence or absence of Neu5Ac. Cells were cultured in medium with (closed circles) or without (open circles) 30 mM Neu5Ac. The values represent the average turbidity (± SD) at 600 nm obtained from three independent biological replicates.

To elucidate if *C*. *perfringens* strain 13 could use Neu5Ac as a nutrient, experiments comparing the growth of the strain in semi-defined medium in the presence or absence of 30 mM Neu5Ac were carried out. As shown in Fig 2, the cells grown with Neu5Ac had a reduced generation time, 50 ± 0.6 min compared to 98 ±12 min (mean ± SD) and increased growth yield (OD_600_ of ca. 0.52 compared to ca. 0.16) in comparison to bacteria grown in the semi-defined medium alone. These results suggest that strain 13 can metabolize Neu5Ac, most likely using the pathway shown in Fig 1B.

### Mutagenesis of *nanI* and *nanJ* indicates NanI activity is induced by the addition of Neu5Ac in *C*. *perfringens* strain 13

**Fig 3:**
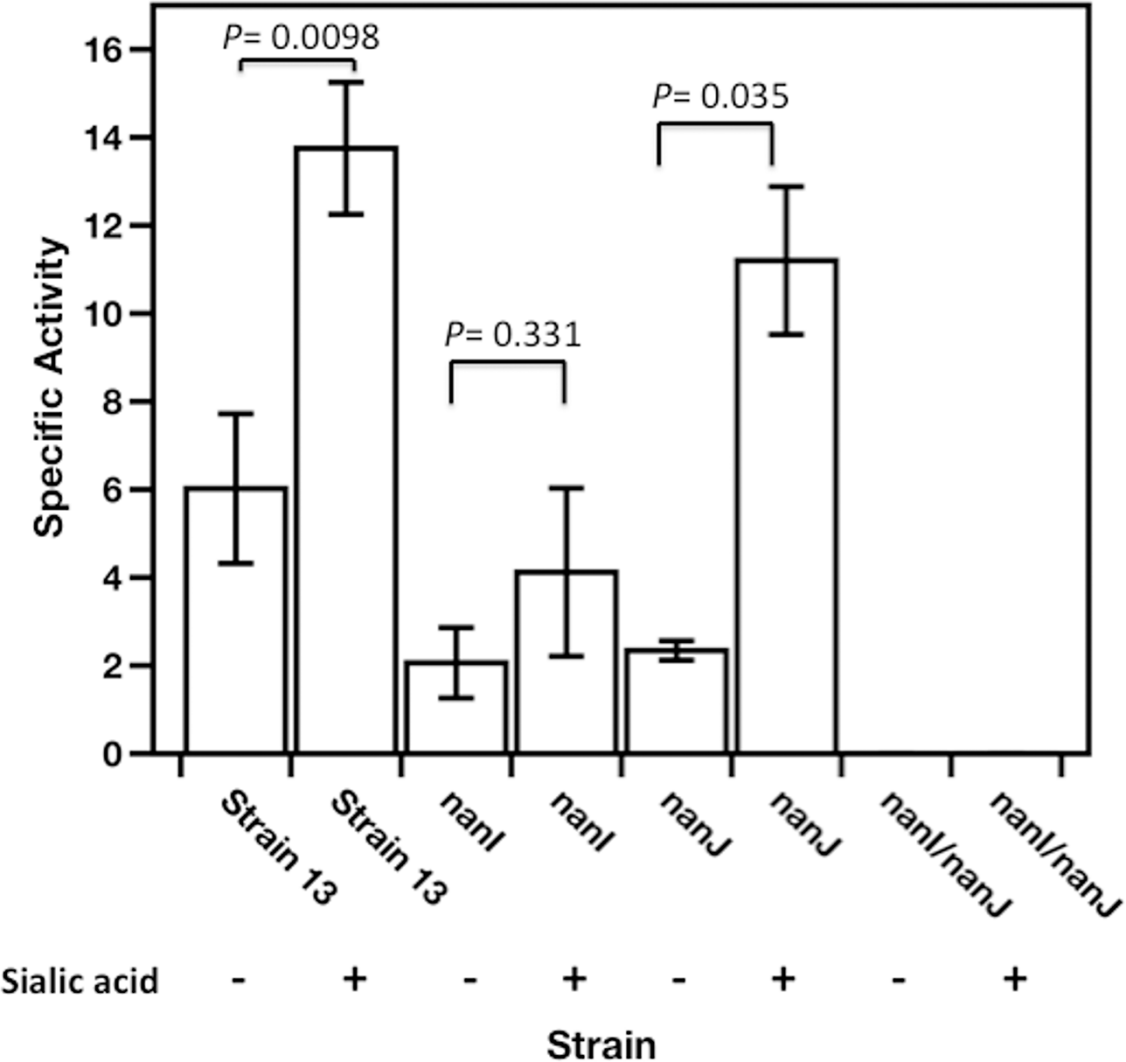
Extracellular sialidase activity of wild-type *C*. *perfringens*, BT1 (*nanI*), BT2 (*nanJ*), and BT3 (*nanInanJ*). Cells were grown overnight in 5 ml of PY with (+) or without (-) 1 mg/ml sialic acid as indicated. Sialidase specific activity was defined as μmoles of 4-methylumbelliferyl-**α**-D-N-acetylneuraminic acid hydrolyzed per minute per milligram of protein. Values shown are the mean and SEM of triplicate biological samples. The *P* values, calculated using the student's two-tailed t-test, are shown in the figure.

To determine if production of the extracellular sialidase enzymes encoded by the *nanI* and *nanJ* genes in strain 13 was inducible by Neu5Ac, we used insertion mutagenesis (i.e., homologous recombination) of internal fragments of the *nanI* and *nanJ* genes in a suicide plasmid to mutate each gene separately and together. The sialidase activity of strain BT1 (*nanI* mutant) was 30% of the wild-type level in the presence of Neu5Ac (Fig 3), and 33% of the activity of the wild-type strain in the absence of Neu5Ac. In comparison to strain 13, strain BT2 (*nanJ* mutant) activity was 81% and 39% in the presence and the absence of Neu5Ac, respectively. Measurement of the sialidase activity of the double mutant BT3 indicated the strain lacked all sialidase activity in the presence or absence of sialic acid (Fig 3). Chiarezza *et al*. [12] showed that NanI was the major sialidase while NanJ was the minor sialidase in strain 13 in the absence of sialic acid. The levels of activity detected in the *nanI* and *nanJ* mutants grown in the absence of sialic acid in this study were actually similar (Fig 3). These differences may be due to the composition of the growth media used in each study, PY here and Todd-Hewitt broth in Chiarezza *et al*. [12]. In the current study the NanI-dependent sialidase activity (i.e., that seen with the *nanJ* mutant) was induced about 4-fold by the addition of Neu5Ac, whereas NanJ-dependent activity (i.e., that seen with the *nanI* mutant) was not statistically different in the presence or absence of Neu5Ac (Fig 3).

### The *nanI* promoter is induced by Neu5Ac

**Fig 4:**
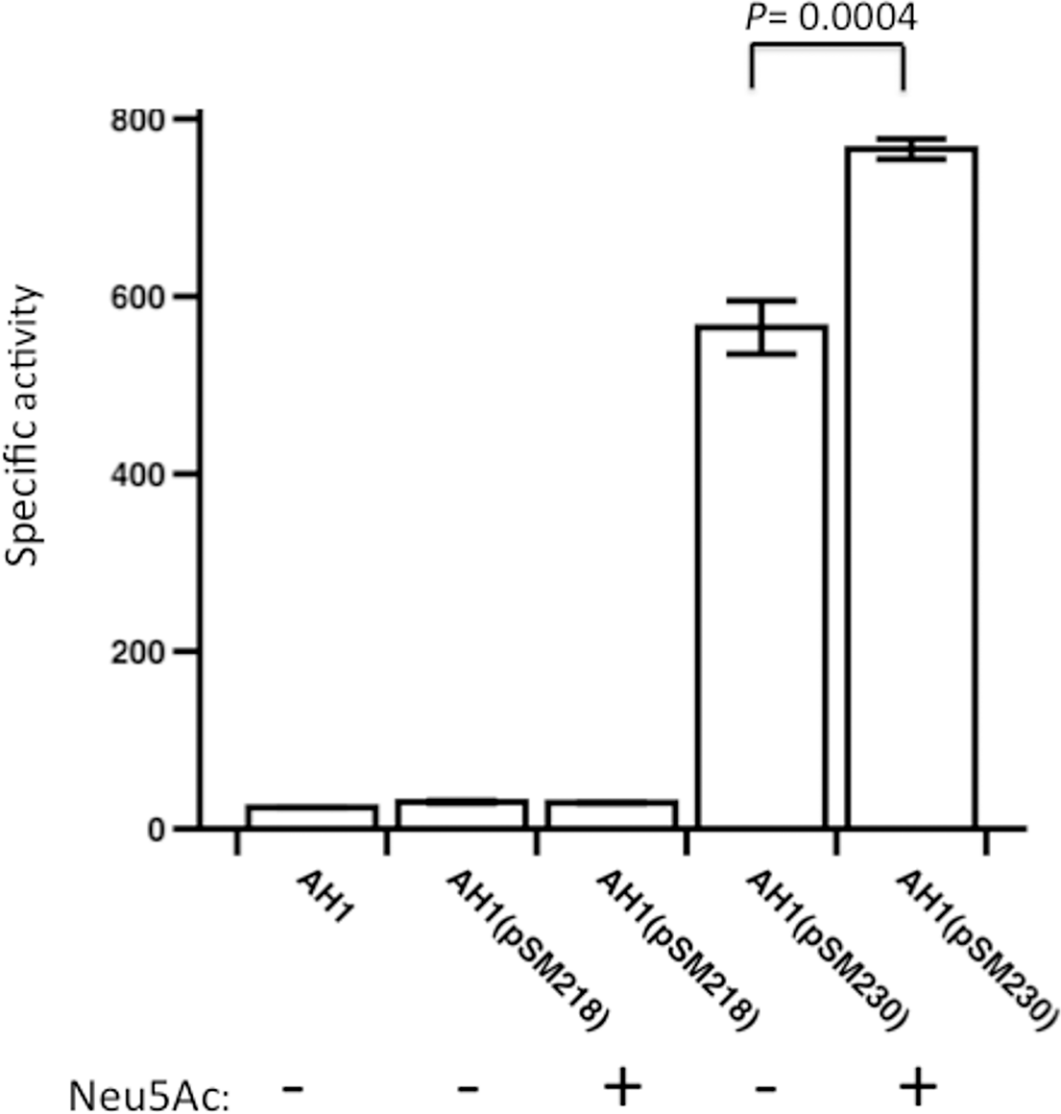
The *nanI* promoter from strain 13(pSM230) shows sialic acid-inducible activity. β-glucuronidase activity was measured for each strain indicated. Strain AH1 is a **β**-glucuronidase mutant derived from strain 13 [31]. Values shown are the mean and SEM of triplicate biological samples. The *P* value, calculated using the student's two-tailed t-test, is shown in the figure.

The intergenic region upstream of the *nanI* gene (i.e., the *nanI* promoter region) was cloned upstream of a promoterless *gusA* gene from *E*. *coli* in the shuttle plasmid pSM218 [14] to give pSM230. Each plasmid was then used to transform *C*. *perfringens* strain AH1, a derivative of strain 13 in which a mutation was introduced in the major **β**-glucuronidase-encoding gene [31]. Strain AH1(pSM218) showed low levels of **β**-glucuronidase activity, while AHI(pSM230) exhibited significantly higher levels of activity (Fig 4). The addition of Neu5Ac to the growth medium stimulated activity from the *nanI* promoter by 35%, similar to what was observed with the *nanE* promoter under similar conditions [14].

### Identification of transcription start sites for *nanI* and *nanJ* by primer extension experiments

**Fig 5:**
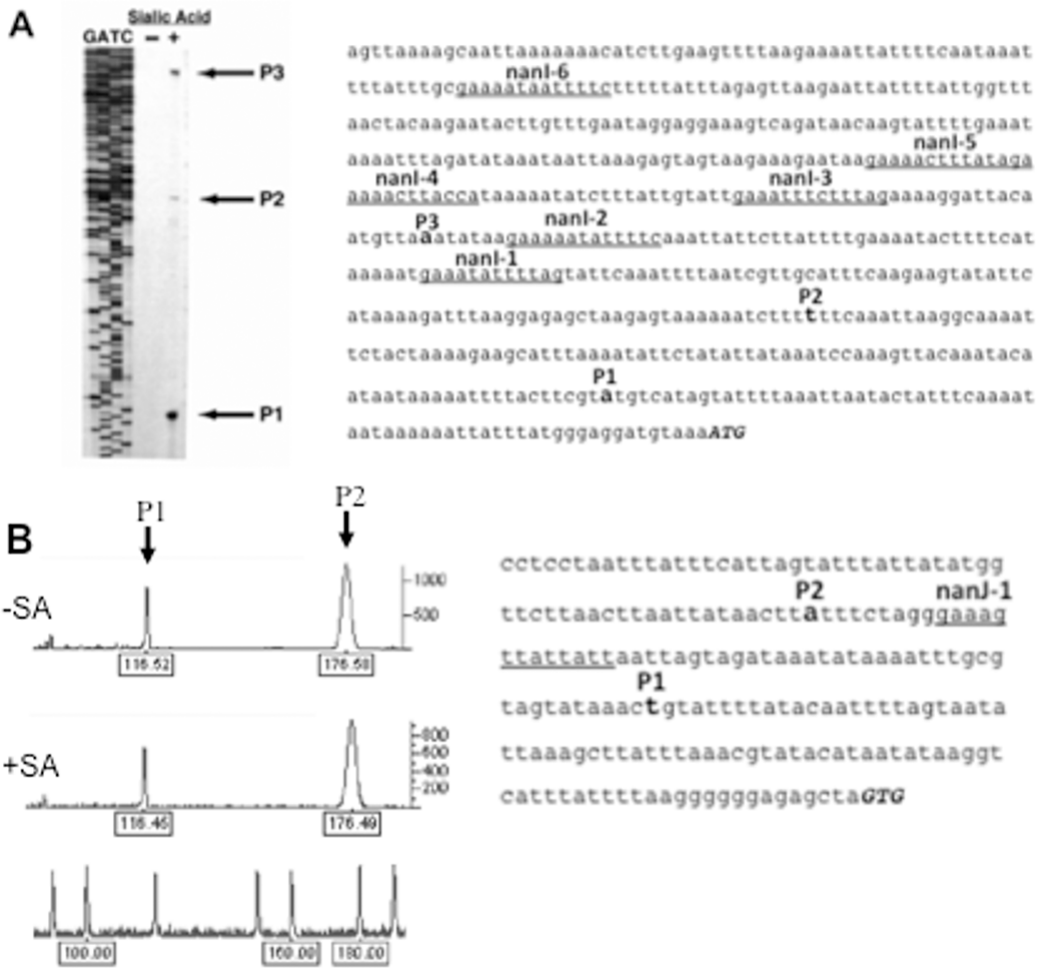
Identification of transcription starts sites upstream of *nanI* and *nanJ*. **(A)**. Primer extension results on RNA isolated from *C*. *perfringens* strain 13 using a primer specific for *nanI* mRNA. The cells were grown on PY (-) or PY plus 1 mg/ml Neu5Ac (+). The GATC lanes show the results of sequencing using the same primer used for the primer extension experiments. The arrows labeled P1, P2, and P3 indicate the position of the putative start sites. The location of P1, P2, P3 and conserved 14 bp elements are shown in the *nanI* promoter sequence. **(B)**. Primer extension results for *nanJ* from *C*. *perfringens* strain 13. The graph shows peak fluorescence of the 5’- 6 FAM labeled RT-PCR product for *nanJ*. Numbers below each peak indicate the length of the product and the peaks on the bottom line show the location of size standards used to estimate the length of the transcripts. The cells were grown on PY (-SA) or PY plus 1 mg/ml Neu5Ac (+SA). The location of P1, P2 and a conserved 14 bp element are shown in the *nanJ* promoter sequence.

To identify the promoters of *nanI* and *nanJ*, primer extension analysis was done with RNA extracted from strain 13 growing with or without Neu5Ac as a carbon source. We identified three 5’ transcription start sites that may represent promoters in the region upstream of the *nanI* gene (Fig 5A). The 5’-ends were located 66, 163, and 308 bp upstream of the start of the *nanI* coding sequence. In addition, all of these transcripts were induced by the presence of Neu5Ac in the growth medium (Fig 5A), suggesting that Neu5Ac acts as an inducer or derepressor of *nanI* transcription.

For *nanJ*, detection of transcripts labeled with the 6-FAM fluorescently tagged primer yielded two peaks, indicating transcripts of 117-bp and 177-bp in length (Fig 5B). These two products indicated that *nanJ* may have two promoters with transcription start sites located 86-bp upstream (P1) and 146-bp upstream (P2) of the *nanJ* start codon (Fig 5B). In contrast to *nanI*, the levels of *nanJ* transcript did not change in the presence of Neu5Ac in the growth medium, as seen in the peak areas of the respective transcripts (Fig 5B).

### NanR binds specifically to the *nanE* and *nanI* promoter regions

**Fig 6:**
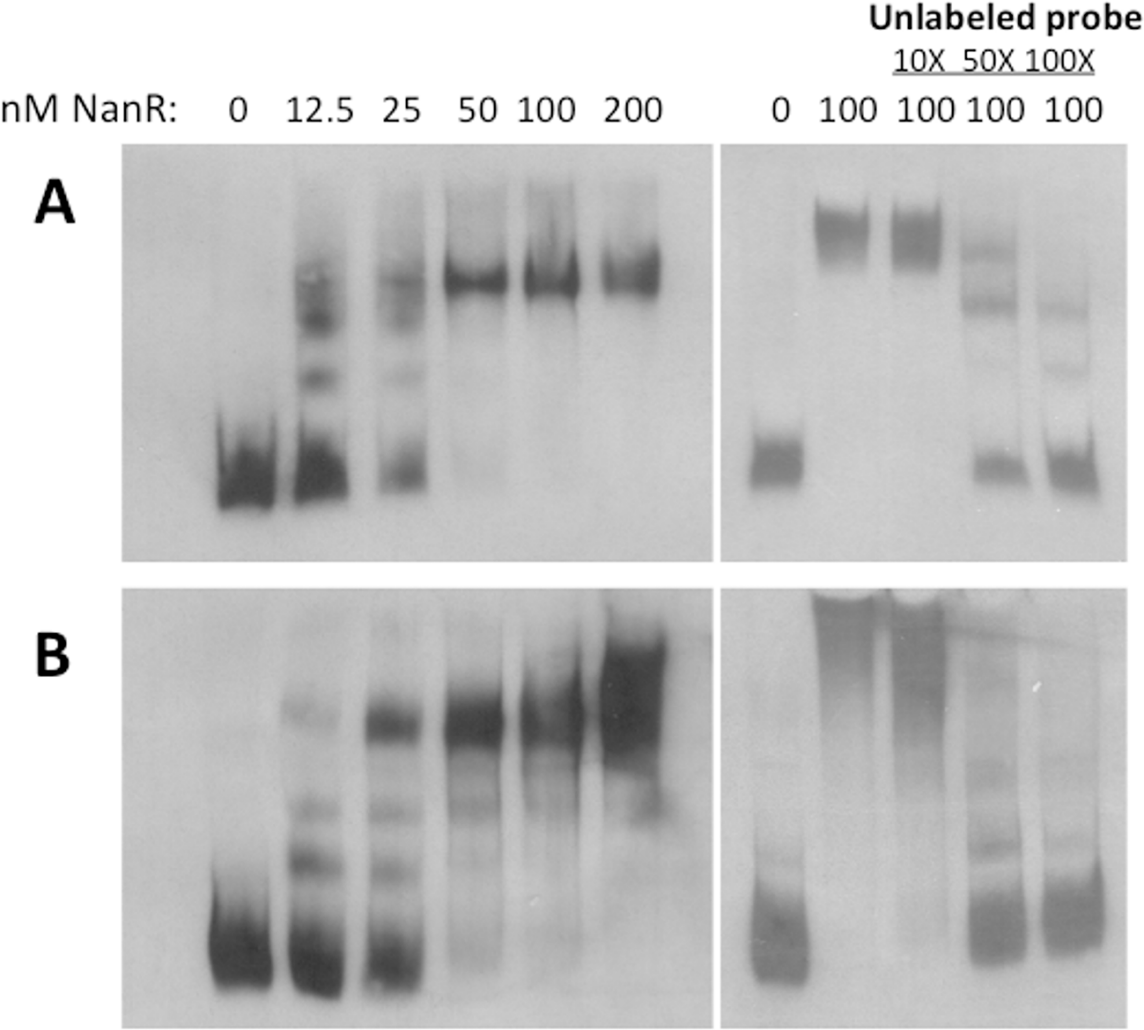
Gel mobility shift experiments with NanR and the *nanI* and *nanE* promoter regions. Gel mobility shift assays with the *nanE* **(A)** and *nanI* **(B)** promoter regions that were PCR amplified as described in the Materials and Methods. The binding reactions shown in panel B were incubated under the same conditions as those in A. In (A) and (B) the panels on the right show the results of binding competition experiments using unlabeled target DNA as indicated.

Gel mobility shift experiments with fragments containing the *nanI* and *nanE* promoter regions indicated that NanR bound with nM-range affinity to these regions (Fig 6A and 6B, left panels). Competition experiments showed that NanR binding to both promoters could be reduced using specific unlabeled DNA (Fig 6A and 6B, right panels), indicating that binding was specific for the *nanI* and *nanE* promoter DNA.

## Discussion

The *nanEAT-yhcH-nanKR* locus and the sialidase genes appear to encode the enzymes needed for production, transport and metabolism of sialic acid to N-acetyl glucosamine (Fig 1), which can then be further metabolized to fructose-6-P for use in glycolysis. Neu5Ac was able to support the growth of *C*. *perfringens* strain 13 in a semi-defined medium, providing evidence that *C*. *perfringens* can use sialic acid as a nutrient source (Fig 2). Since sialic acid is a component of mucin and other polysaccharides found in mammals [39], free sialic acid would be liberated by the action of extracellular sialidases and be transported into and metabolized by *C*. *perfringens* in the intestinal tract or during the course of tissue infections. In support of this model, *C*. *perfringens* sialidases have been shown to liberate free sialic acid from mammalian cells *in vitro* [40]. However, since a mutant lacking both sialidases was still virulent in a mouse myonecrosis model [12] it suggests that Neu5Ac metabolism is not essential for growth *in vivo*.

Comparison of the sialidase activity of *nanI* and *nanJ* mutants of strain 13 showed that NanI accounts for about 2/3 of the total activity in the presence of sialic acid (Fig 3). These results are in agreement with previous data that showed that NanI was the major sialidase produced by strain 13 [12]. Similar results were found with the levels of secreted sialidase activity by the type D strain, CN3178, which, unlike strain 13, produces the intracellular sialidase NanH [40]. For strain 13, the NanI-dependent sialidase activity was induced about 4-fold by the addition of Neu5Ac, while NanJ-dependent activity was not significantly induced by the addition of Neu5Ac.

Using primer extension analysis, we were able to identify three putative transcription start sites in the *nanI* promoter region (Fig 5A). In a previous report [21], multiple transcription start sites were identified upstream of the *nanI* gene in strain 13. The site that these authors considered as the major site that responded to the presence of the regulatory proteins CPE1446-CPE1447 matches the P2 transcript shown in Fig 5A. P1 and P3 were not identified as transcripts in the previous study, which may be due to the differences in growth conditions used in the two studies, rich medium (Gifu anaerobic medium (GAM broth)) in [21] and PY medium here. Each of the putative promoters we identified was also inducible by the presence of 1 mg/ml Neu5Ac in the growth medium. Fusions of the *nanI* promoter region to the *gusA* gene of *E*. *coli* provided further evidence that the promoters were induced by Neu5Ac (Fig 4). The two *nanJ* transcripts we identified by primer extension analysis were not the same as the single transcript reported previously [20], which again may be due to the differences in growth conditions resulting from the use of GAM broth in [20] and PY medium here. The two *nanJ* transcripts we identified were not induced by Neu5Ac (Fig 5), suggesting that *nanJ* expression is not regulated by Neu5Ac.

Identification of -10 and -35 **σ** ^A^ sigma factor recognition sequences in *C*. *perfringens* is complicated by the high level (>80%) of A-T bases found in intergenic regions [8, 11], which are also found in consensus Gram-positive -10 and -35 elements, TATAAT and TTGACA, respectively [41]. Analysis of the sequences upstream of the three transcription start sites for *nanI* and the two for *nanJ* show 5/6 bases match the consensus for either -10 or -35 regions, but none have good matches to both. This may indicate additional transcription regulators are needed or other sigma factors are involved in transcribing these genes.

The NanR protein is predicted to be a member of the RpiR transcriptional regulator family, based on its low level of sequence identity (23.8 to 27.2% identity) to other RpiR family proteins involved in regulating sialic acid metabolism, including those from *S*. *aureus*, *S*. *pneumoniae* and *Vibrio vulnificus*. We purified the NanR protein and were able to demonstrate high affinity (in the range of 12.5–25 nM) and specific binding to the *nanE* and *nanI* promoter regions (Fig 6). Gel mobility shift experiments with purified NanR and the regions upstream of the *nanJ* and *nanR* genes showed very weak binding to these regions at the higher NanR concentrations under our experimental conditions (S3 Fig in Supplemental Information).

**Fig 7:**
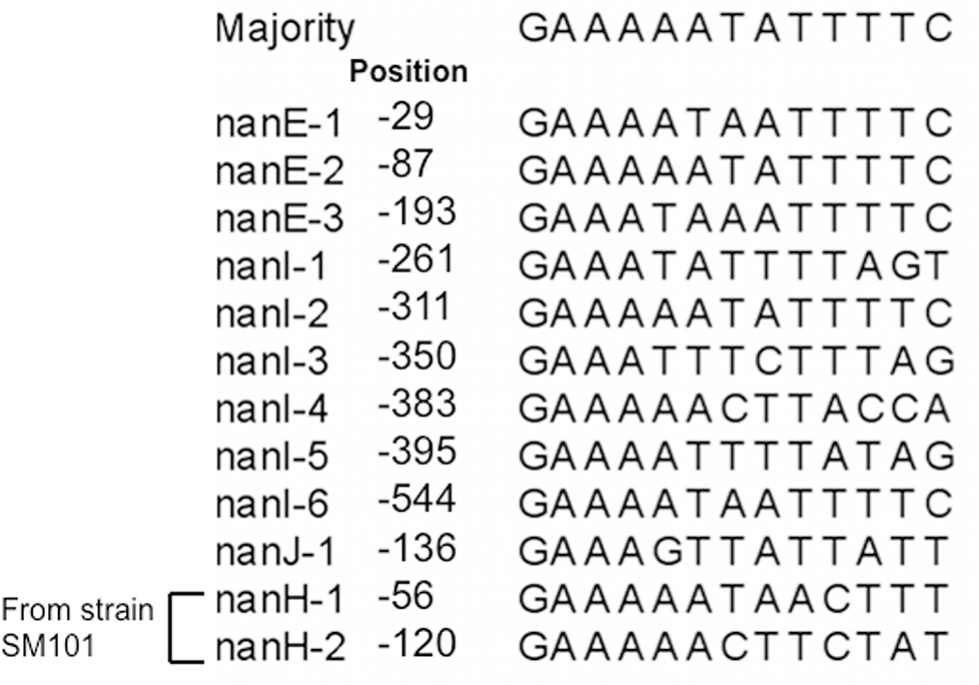
Alignment of conserved 14-bp elements located in the promoter regions of sialic acid-related genes. Majority refers to the consensus sequence calculated by the Megalign program (Lasergene, v. 11, DNASTAR). “Position” refers to the distance from the adenosine in the initiator methionine residue for each gene shown to the left. The numbers following each gene indicate the proximity of the sequence to the start codon with the sequences closest to the start represented by the lowest number.

We examined the sequences of the promoter regions of sialic acid-associated genes to determine if there were any conserved sequence elements and discovered a conserved 14 bp sequence (consensus sequence of 5’-GAAAAATATTTTC-3’) that was present in the promoter regions of *nanI*, *nanE*, *nanJ*, and the putative promoter of *nanH* from strain SM101 (Fig 7). These conserved repeats are in regions known to be the start sites of transcription for these promoters (Fig 5 and [14]), suggesting that they may function as a recognition sequence for a transcriptional regulator, possibly NanR or one of the other proteins shown to regulate sialidase production in *C*. *perfringens*. The single element found in the *nanJ* promoter (Fig 5B and Fig 7) differs from the consensus at 4 bp, possibly explaining why strong binding by NanR was not observed with this DNA fragment and why Neu5Ac did not stimulate enzyme synthesis (Fig 3). In a recent report, a putative NanR binding site was identified in *S*. *pneumoniae* by sequence conservation and promoter truncation experiments [42]. The consensus NanR binding site was an 18-bp palindromic sequence (5'-TCTGAAASTACTTTCARA-3') which shows some similarity to the 14 bp conserved sequence we identified in *C*. *perfringens* (5’-GAAAAATATTTTC-3’), suggesting there may be conserved sequence recognition by Gram positive NanR proteins.

**Fig 8:**
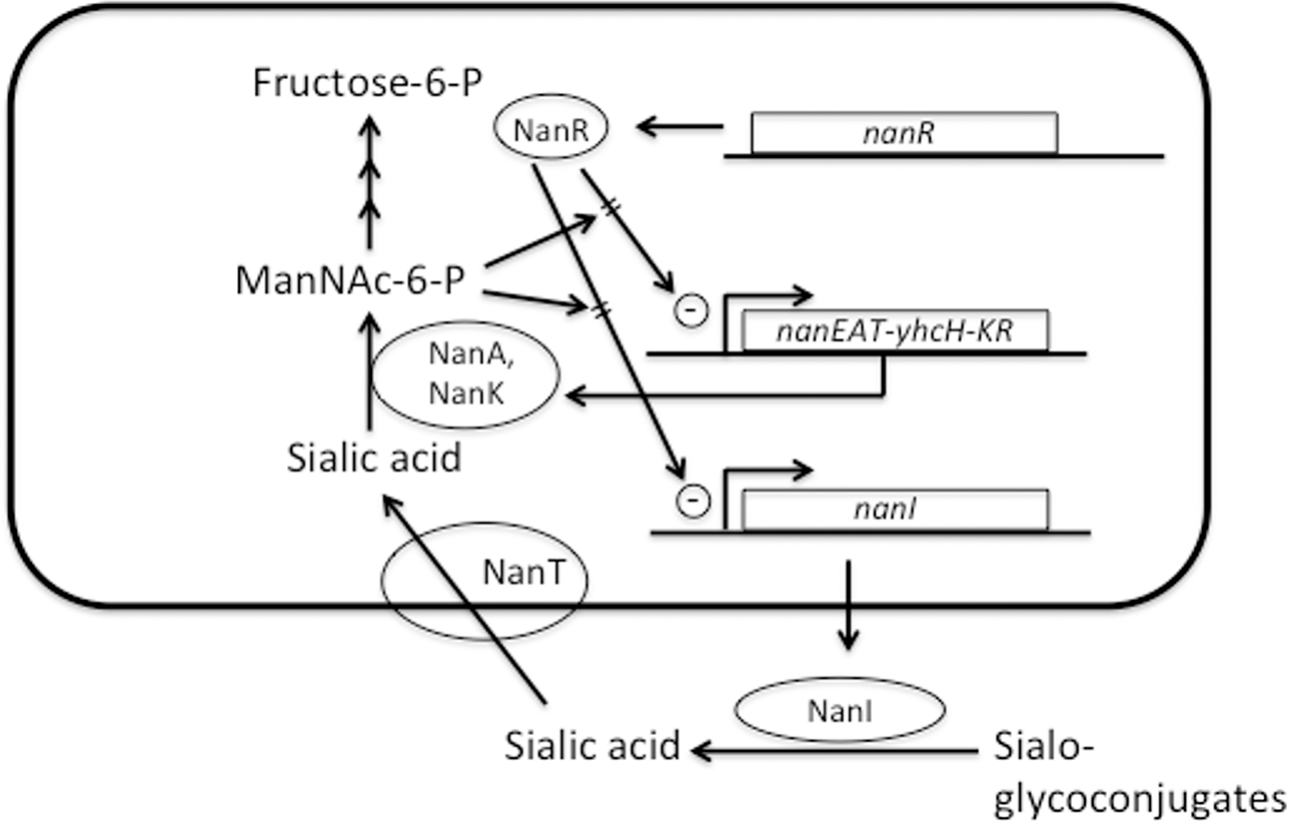
Model showing the operation of a positive feedback loop for *nanI* and *nanEAT-yhcH-nanKR in vivo*. See text for detailed description of the model features.

The NanR regulator is postulated to be at the center of a positive feedback loop that responds to the presence of sialic acid (Fig 8). In this model, secreted NanI acts on host sialoglycoconjugates to produce free sialic acid, which is then transported into the cell and metabolized. We suggest that *C*. *perfringens* NanR, like its homologs in *S*. *aureus* and *V*. *vulnificus* [25], binds to the intermediate ManNac-6-P, which acts to relieve transcriptional repression on the *nanE* and *nanI* operators, leading to increased NanI synthesis and secretion, more free sialic acid, and more ManNac-6-P. By using ManNac-6-P as the molecular signal, the pathway would ensure that free sialic acid is released, transported and metabolized by the products of the NanA and NanK enzymes and that the complete metabolic pathway is operational. Overlaying this positive feedback loop are the VirSR, RevR, Tex, CPE1446-CPE1447 and ReeS regulatory systems, all of which have been shown to be involved in the regulation of *nanI* and *nanJ*, and for VirSR and RevR, the *nanEAT*-*yhcH*-*nanKR* operon [17].

Finally, there is the question as to what is the functional role of NanJ in an infection. Analysis of NanJ activity in the type D strain CN3718 revealed a different preference for sialic acid linkages compared to NanI from the same strain [40]. NanJ has several domains that are not present in NanI [43]. At least two of these domains are involved in binding carbohydrates containing galactose/N-acetylgalactosamine and sialic acid, leading other workers [43] to postulate that NanJ may function as a broad specificity polysaccharide binding protein that can liberate sialic acid from a variety of sialoglycoconjugates. If true, one consequence would be the production of free sialic acid from a variety of substrates, which would then act as an activator for *nanI* transcription and synthesis of the NanI protein and the enzymes involved in sialic acid metabolism. In this scenario, NanJ functions as a signal synthesis enzyme that leads to activation of NanI. Further experiments designed to measure the levels of NanI and NanJ in tissues during the course of a gangrene infection are needed to resolve this issue.

## Supporting Information

S1 Fig Southern blot analysis of sialidase gene mutations. (A). Chromosomal DNA from strain 13 (lane 1) and the nanI mutant strain (lane 2) were hybridized with a nanI-specific probe. (B). Chromosomal DNA from strain 13 (lane 1) and the nanJ mutant strain (lane 2) were hybridized with a nanJ-specific probe. (C). Chromosomal DNA from strain 13 (lane 1) and the nanI/nanJ mutant strain (lane 2) were hybridized with a nanJ-specific probe. DNA size markers are shown to the left of each image and asterisks denote the location of the expected band size in the wild type strain. The bands marked as >10 kb represent the multimeric form of the plasmid used for insertion mutagenesis. For panels B and C, the same probe was used to detect the change in size of the nanJ gene after insertion of the recombinant plasmid.
(PDF)

S2 Fig Level of purity of NanR after purification. Lanes 1–5: Successive fractions eluted from an S200 gel filtration column. Note the presence of a single band in each fraction. The numbers on the left indicate the positions of protein molecular size markers in kDa.
(PDF)

S3 Fig Gel mobility shift experiments with NanR and the nanJ and nanR promoter regions. Gel mobility shift assays with the nanJ (left panel) and nanR (right panel) promoter regions that were PCR amplified as described in the Materials and Methods of the main text.
(PDF)

S1 Text Figure Legends and Methods for Supporting Information. (PDF)
