# Supplementary figures and images for: NanR, a Transcriptional Regulator That Binds to the Promoters of Genes Involved in Sialic Acid Metabolism in the Anaerobic Pathogen Clostridium perfringens
Source: PLoS One. 2015 Jul 21;10(7):e0133217. doi: 10.1371/journal.pone.0133217 (PMC4509764; doi:10.1371/journal.pone.0133217)

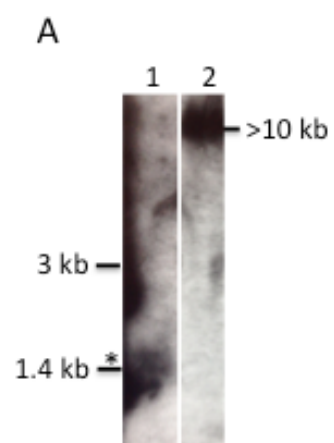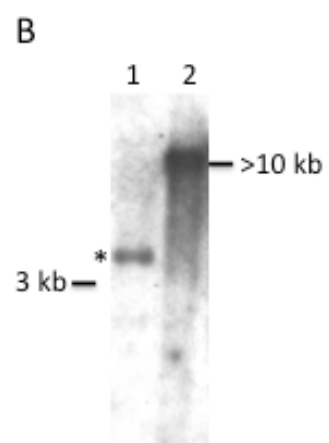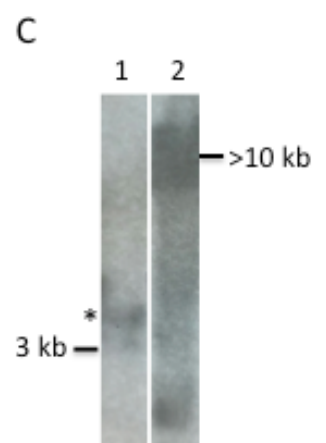

Supplement: S1 Fig — (A). Chromosomal DNA from strain 13 (lane 1) and the nanI mutant strain (lane 2) were hybridized with a nanI-specific probe. (B). Chromosomal DNA from strain 13 (lane 1) and the nanJ mutant strain (lane 2) were hybridized with a nanJ-specific probe. (C). Chromosomal DNA from strain 13 (lane 1) and the nanI/nanJ mutant strain (lane 2) were hybridized with a nanJ-specific probe. DNA size markers are shown to the left of each image and asterisks denote the location of the expected band size in the wild type strain. The bands marked as >10 kb represent the multimeric form of the plasmid used for insertion mutagenesis. For panels B and C, the same probe was used to detect the change in size of the nanJ gene after insertion of the recombinant plasmid. (PDF) [file pone.0133217.s001.pdf]

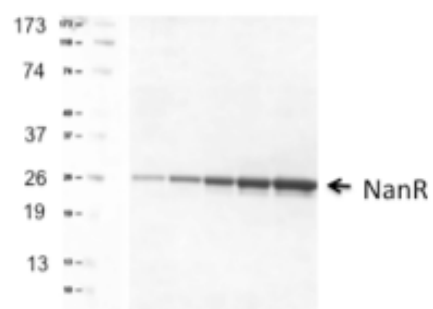

Supplement: S2 Fig — Lanes 1–5: Successive fractions eluted from an S200 gel filtration column. Note the presence of a single band in each fraction. The numbers on the left indicate the positions of protein molecular size markers in kDa. (PDF) [file pone.0133217.s002.pdf]

## Gel Mobility Shift Experiments

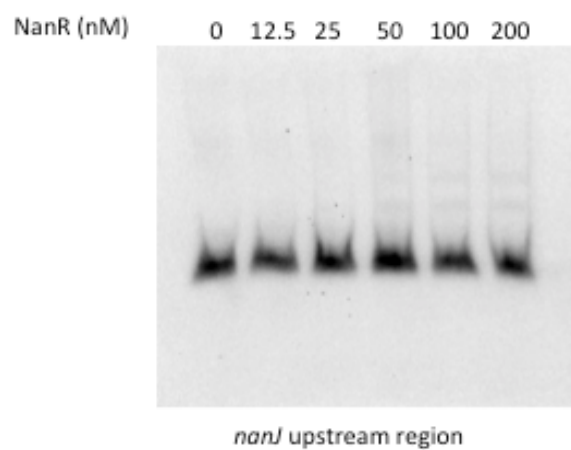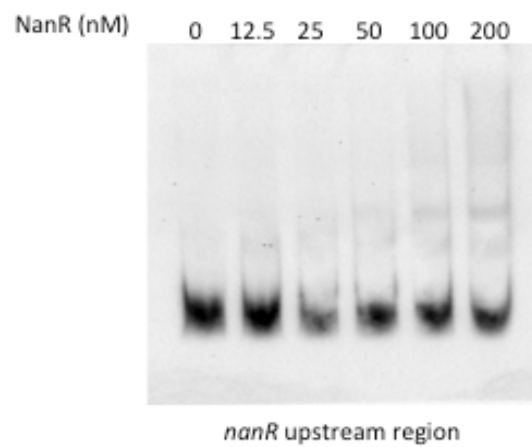

Supplement: S3 Fig — Gel mobility shift assays with the nanJ (left panel) and nanR (right panel) promoter regions that were PCR amplified as described in the Materials and Methods of the main text. (PDF) [file pone.0133217.s003.pdf]
