# Supplementary material for: NanR, a Transcriptional Regulator That Binds to the Promoters of Genes Involved in Sialic Acid Metabolism in the Anaerobic Pathogen Clostridium perfringens
Source: PLoS One. 2015 Jul 21;10(7):e0133217. doi: 10.1371/journal.pone.0133217 (PMC4509764; doi:10.1371/journal.pone.0133217)
Supplement: S1 Text — (PDF) [file pone.0133217.s004.pdf]

Figure Legends for Supplemental Material

Fig. S1. Southern blot analysis of sialidase gene mutations. (A). Chromosomal DNA from strain 13 (lane 1) and the *nanI* mutant strain (lane 2) were hybridized with a *nanI*-specific probe. **(B).** Chromosomal DNA from strain 13 (lane 1) and the *nanJ* mutant strain (lane 2) were hybridized with a *nanJ*-specific probe. **(C).** Chromosomal DNA from strain 13 (lane 1) and the *nanI*/*nanJ* mutant strain (lane 2) were hybridized with a *nanJ*-specific probe. DNA size markers are shown to the left of each image and asterisks denote the location of the expected band size in the wild type strain. The bands marked as >10 kb represent the multimeric form of the plasmid used for insertion mutagenesis. For panels B and C, the same probe was used to detect the change in size of the *nanJ* gene after insertion of the recombinant plasmid.

Fig. S2. Level of purity of NanR after purification. Lanes 1-5: Successive fractions eluted from an S200 gel filtration column. Note the presence of a single band in each fraction. The numbers on the left indicate the positions of protein molecular size markers in kDa.

Fig. S3. Gel mobility shift experiments with NanR and the *nanJ* and *nanR* promoter regions. Gel mobility shift assays with the *nanJ* (left panel) and *nanR* (right panel) promoter regions that were PCR amplified as described in the Materials and Methods of the main text.

Material and Methods for Supplemental Material

Southern blots. Chromosomal DNA was isolated using the method of Mengaud *et al* [1], digested with *Nde*I for *nanI* detection and *Sca*I for *nanJ* detection. The digested DNA was loaded onto agarose gels for electrophoresis and subsequently transferred to a nylon membrane (Schleicher and Schuell) according to a previously described method [2]. The probes for *nanI* and *nanJ* were made by restriction digestion of pDOB18 and pBT5 to release the *nanI* and *nanJ* gene fragments, purification of the fragments from agarose gels, followed by labeling with biotinylated nucleotides using the NEBlot Kit from New England Biolabs, based on the manufacturer's procedures. Hybridization and detection were done using the Phototope-Star Detection Kit (New England Biolabs) and X-ray film, according to the manufacturer's protocol. Based on restriction maps of the respective genes, the wild type should show hybridization of the probes to a band at 1.4 kb for *nanI* and 3.9 kb for *nanJ*. Since the *nanI* mutant was the host for *nanJ* mutagenesis, to construct the *nanI*/*nanJ* strain, only the *nanJ* probe was used for detecting the mutation.

References for Supplemental Materials

1. Mengaud J, Geoffroy C, Cossart P (1991) Identification of a new operon involved in *Listeria monocytogenes* virulence: its first gene encodes a protein homologous to bacterial metalloproteases. Infect Immun **59**: 1043-1049.

2. Sambrook J, Russel DW (2001) Molecular Cloning, A Laboratory Manual, 3rd Edition. Cold Spring Harbor, New York: Cold Spring Harbor Laboratory Press.
